# Analysis of the global and local effects of urban green space vegetation types on early-summer near-surface O_3_ concentrations based on street view imagery – A case study of hangzhou

**DOI:** 10.1371/journal.pone.0358563

**Published:** 2026-10-27

**Authors:** Meng Tang, Min Li

**Affiliations:** 1 College of Landscape Architecture, Nanjing Forestry University, Nanjing, Jiangsu, China; 2 School of Architecture, South China University of Technology, Guangzhou, Guangdong, China; Zhejiang A and F University, CHINA

## Abstract

With the expansion of urban development, air pollution has become increasingly severe. In recent years, ozone (O_3_) pollution has emerged as a major threat to air quality in China. Existing studies have rarely explored the impact of vegetation types on O_3_ from a micro-scale perspective. Taking Hangzhou, a city with severe O_3_ pollution, as an example, street-view images from eight districts were first collected. A DeepLabV3+ semantic segmentation model trained on the ADE20K dataset was employed to accurately classify trees, shrubs, and grasses in the images. Meteorological and urban structural factors were incorporated, and Bayesian-optimized Optimization-based Extreme Gradient Boosting (XGBoost), SHAP (SHapley Additive exPlanations), and Geographically Weighted Random Forest (GWRF) models were applied to analyze the independent effects of the three vegetation-type factors on O_3_ concentration and their synergistic interactions from both global and local scales. The results indicate: (1) Xiaoshan District and the core urban areas such as Xihu District and are severely polluted, with an overall decreasing trend in O_3_ concentration (the area with O_3_ concentration in the range of 120.01–160.00 μg/m^3^ decreased from 2,100 km^2^ to 0 km^2^). (2) Meteorological factors dominate O_3_ variation, while the contributions of vegetation-type factors are relatively low. Nevertheless, vegetation-type factors still exhibit stable and differentiated contributions to O_3_ concentration. At the global scale, tree area proportion (TAP) shows either positive or negative effects on O_3_ concentration at high levels (average contribution = 0.002), shrub area proportion (SAP) has a positive influence, and grass area proportion (GAP) demonstrates a negative influence (average contribution = 0.006 for both). (3) At the local scale, the extremely high-importance areas of TAP shifted from northern Xihu District and Gongshu District in 2014 to southern Xihu District and Binjiang District in 2017. SAP exhibited relatively high and extremely high importance in southern Xihu District and western Xiaoshan District. The relatively high and extremely high importance areas of GAP were distributed in core urban zones such as eastern Xihu District, southern Gongshu District, Binjiang District, and Shangcheng District. The extent of extremely high-importance areas for all vegetation-type factors showed a decreasing trend. (4) High-importance areas of building density (BD) and industrial impervious surface percentage (ISP) spatially overlapped with those of vegetation-type factors, concentrating in core urban areas and the industrial zone of Xiaoshan, whereas meteorological factors played an independent role mainly in the peripheral areas of the city. This study aims to provide a scientific basis for precise O_3_ pollution control and optimal configuration of urban green spaces.

## 1. Introduction

Cities are the primary areas for human habitation and activities, where pollutants are continuously emitted into the atmosphere through domestic heating, vehicular traffic, industrial production, and other anthropogenic activities [1,2]. Relevant studies indicate that, as of 2023, urban areas account for approximately two-thirds of global energy consumption and contribute to about 70% of global greenhouse gas emissions [3]. Air pollution has long been recognized as one of the most severe environmental issues affecting human health.

Fine particulate matter (PM2.5) and O_3_ are key constituents of atmospheric pollutants [4]. In recent years, near-surface O_3_ has emerged as a primary threat to air quality in China. Overall, O_3_ pollution has even surpassed PM2.5 pollution, becoming the dominant air pollutant in the country [5]. Long-term exposure to O_3_ concentrations exceeding monitoring standards can lead to increased incidence of respiratory and cardiovascular diseases—such as emphysema, asthma, and lung cancer—and elevate mortality risk [6]. Moreover, O_3_ poses significant threats to terrestrial ecosystems and biodiversity. Experimental findings indicate that O_3_ exerts toxic effects on plants, inhibiting photosynthesis and reducing evapotranspiration capacity [7]. Prolonged exposure to high O_3_ concentrations can alter ecosystem carbon allocation and diminish vegetation productivity [8]. Consequently, scientifically understanding the dynamics of urban O_3_ pollution and achieving effective regulation of its concentrations are critical issues that must be urgently addressed.

Vegetation within urban green spaces provides ecosystem services in the urban environment and can help mitigate O_3_ pollution. Plant community structure and diversity influence the effectiveness of O_3_ pollution alleviation [6]. Notably, vegetation also emits biogenic volatile organic compounds (BVOCs), which serve as precursors for O_3_ formation. High emissions of BVOCs can lead to significant increases in surface-level O_3_ and facilitate its transport and diffusion. Different vegetation types vary considerably in their BVOCs emissions [6], thereby affecting O_3_ concentrations. In terms of research focus, existing studies on the impact of green spaces on ambient O_3_ pollution remain limited, with most research concentrating on vegetation’s effects on particulate pollutants (PM2.5 and PM10) [9–11]. Moreover, macro-level factors such as green space area [12], spatial pattern [13,14], spatial connectivity [15], and vegetation coverage density [16] have frequently been used to explore the relationship between green spaces and air pollution, while micro-level factors like vegetation structure [17] and vegetation type [16,18] have received less attention. Regarding data acquisition, obtaining vegetation type and structure data still relies primarily on traditional field measurements. Although this approach ensures data accuracy, it requires substantial investment of manpower, material resources, and time, often limiting the scope and sample size of studies. Street view imagery, as an emerging source of geospatial data, offers advantages such as extensive coverage, high acquisition efficiency, and alignment with pedestrian perspectives [19], and has gradually become an important method to compensate for the limitations of field measurements [20,21]. In terms of research methodology, previous studies have predominantly used linear regression models [22,23] or meteorological and landscape simulation models [2,24] to examine the relationship between vegetation indicators and air pollution indicators. These models suffer from drawbacks such as neglecting nonlinear relationships among variables and limited generalizability, making it difficult to accurately quantify the relationship between vegetation and pollutants [25]. Additionally, some models require complex parameter calibration and high data quality, often relying on precise field measurement data as input, which entails significant human and material resources [26]. Nonlinear tree-based ensemble models such as Optimization-based Extreme Gradient Boosting (XGBoost) and Random Forest (RF) offer flexible model structures and nonlinear function approximation capabilities [27], enabling the capture and modeling of complex variations and handling intricate nonlinear relationships, while also demonstrating strong robustness to multicollinearity [28].

Based on this, this study selects Hangzhou, a city with severe O_3_ pollution in early summer, as the research area [29]. By applying semantic segmentation to street-view images, classification data of trees, shrubs, and grasses are obtained, and the area proportions of these three vegetation types are used as indicators to explore their relationship with O_3_ concentration. The study aims to address three scientific questions: (1) How did the early-summer O_3_ concentration in Hangzhou changes from 2014 to 2020? (2) How can the independent influence of vegetation types such as trees, shrubs, and grass on O_3_ concentration be quantified from a street-view perspective after separating the interference of meteorological and urban structural factors? (3) What are the global and local mechanisms through which vegetation types in urban green spaces affect O_3_ concentration?

## 2. Materials and methods

### 2.1. Study area

Hangzhou is the second-largest city in the Yangtze River Delta region, with a population exceeding 9 million. In recent years, air pollution in Hangzhou has become increasingly severe. According to the Annual Report on Ecological and Environmental Status of Hangzhou, there were 59 days in 2018 when the O_3_ level exceeded the national standard of 160 μg/m^3^, and the 90th percentile of the maximum daily 8-hour average O_3_ concentration reached 170 μg/m^3^ [30]. This phenomenon has drawn attention to O_3_ pollution in Hangzhou. Additionally, Hangzhou has made steady progress in urban greening, with its green coverage rate stabilizing at around 40% [31]. Against this backdrop of extensive green space, O_3_ air pollution remains notably severe in the city’s main urban areas, providing a representative case for related research. Therefore, this study selects eight main urban districts—Binjiang, Gongshu, Linping, Qiantang, Xiaoshan, Yuhang, Shangcheng, and Xihu—as the research areas to investigate the mechanisms through which vegetation types (Fig 1), in synergy with other factors, influence O_3_ air pollution.

**Fig 1:**
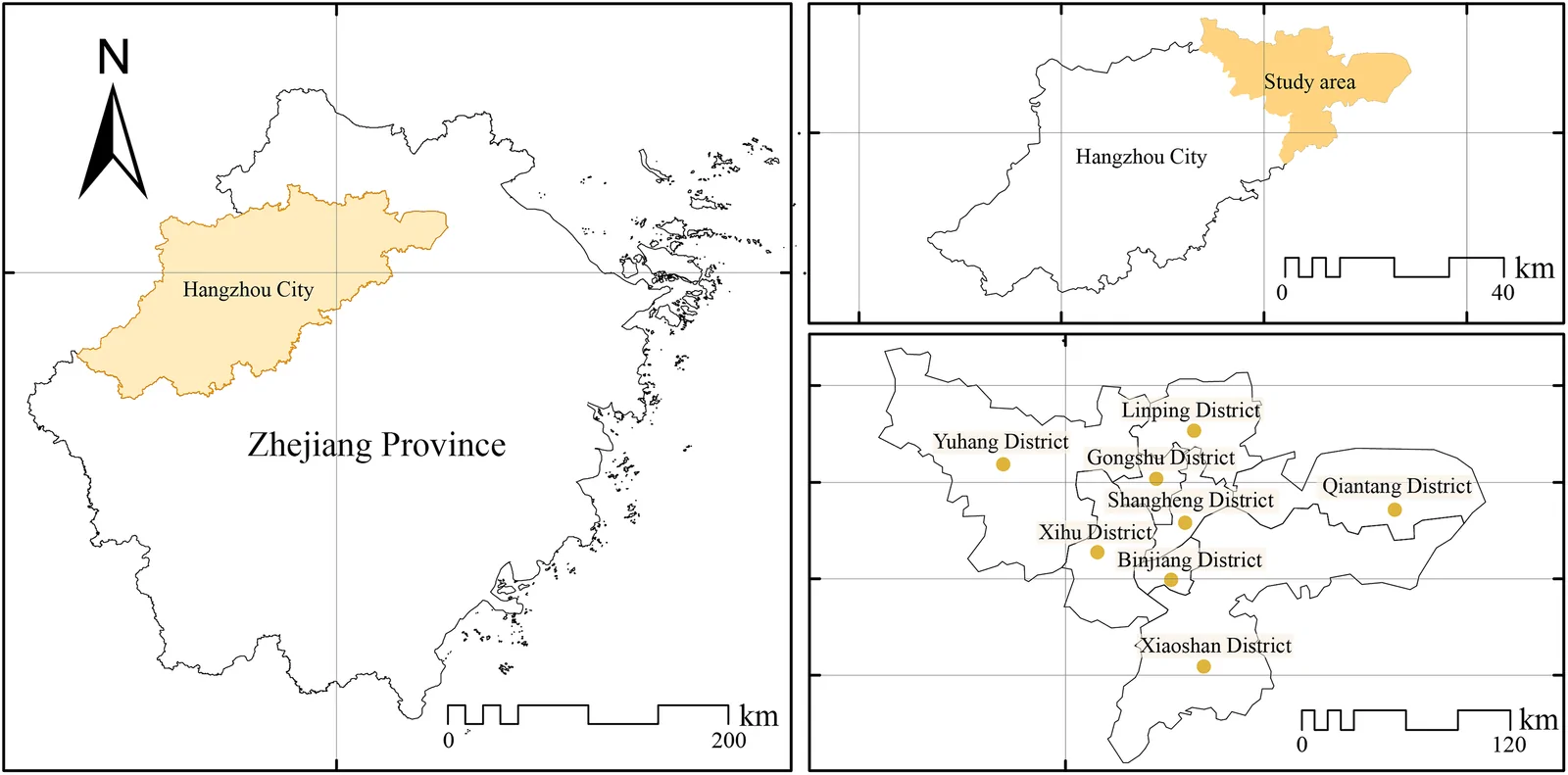
Location map of the study area. (Reference base map from the Zhejiang province map of the standard map service system, Map Approval No.: Zhejiang S (2025) No. 23).

### 2.2. Data sources and processing

Daily near-surface O_3_ concentration data were obtained from the ChinaHighO_3_: High-resolution and High-quality Ground-level MDA8 O_3_ Dataset for China (2000–2023) published by Wei et al. [32] on the National Tibetan Plateau Data Center. This dataset was generated by integrating big data sources such as ground-based observations, atmospheric reanalysis, and emission inventories. It was validated using 10-fold cross-validation against data from more than 1,000 ground monitoring stations nationwide, achieving a coefficient of determination (R^2^) of 0.89 and a root mean square error (RMSE) of 15.77 µg/m^3^, demonstrating high reliability. The dataset has already been used in multiple studies [33,34]. Monthly O_3_ concentrations used in this study were derived by calculating the sum and average of daily raster data for each month. Street-view imagery was acquired via the API of Baidu maps open platform and served as the fundamental data source for vegetation type classification. Meteorological data were retrieved from ChinaMet: A High-resolution Multi-element Meteorological Driving Dataset for China via Multi-source Data Fusion, released by Zhang et al. [35,36] on the National Cryosphere Desert Data Center. This dataset includes near-surface wind speed at 10 m, near-surface average air temperature at 2 m, relative humidity, precipitation, and other variables. Urban structural data were collected from open-access platforms, including OpenStreetMap and the Zenodo repository. Specifically, nighttime light data [37] were obtained from the Reconstructed Three-decade Global Fine-Grained Nighttime Light Dataset, and land cover data [38] from The 30 m annual land cover datasets and its dynamics in China from 1985 to 2024, both hosted on Zenodo. All data collection and analytical procedures in this study strictly comply with the terms of use and license agreements of the respective data sources. The use of Zenodo datasets conforms to its open-access licensing requirements (Table 1).

**Table 1:** Data resources.

Data type	Data name	Spatial resolution	Data source	Data processing
**O**_**3**_ **concentration**	ChinaHighO_3_: High-resolution and High-quality Ground-level MDA8 O_3_ Dataset for China (2000–2023)	1km	National Tibetan Plateau Data Center https://data.tpdc.ac.cn/zh-hans/data/87753867-77c8-42f1-b2e6-da569679635f	The daily O_3_ concentration data were processed using raster calculation in ArcGIS. Monthly average O_3_ concentration data were derived through summation and averaging operations, completing the conversion to monthly O_3_ concentration.
**Street view**	Street view imagery	–	Baidu maps open platform https://lbsyun.baidu.com/	The road network data of the study area were imported into ArcGIS. Street view sampling points were generated at 50-meter intervals, and street view images were batch-acquired via the API interface of Baidu Maps Open Platform and Python programs.
**Meteorology**	ChinaMet: A High-resolution Multi-element Meteorological Driving Dataset for China via Multi-source Data Fusion	1000m	National Cryosphere Desert Data https://www.ncdc.ac.cn/portal/metadata/21691d03-bef2-4800-924e-5614e7268b87	The “Make NetCDF Raster Layer” tool in ArcGIS was employed to export the data to TIFF format. The raster data in TIFF format were clipped to the study area, and raster value assignment to the fishnet grid was performed.
**Urban structure**	Vector building data	1000m	OpenStreetMap https://openstreetmap.us/	After exporting data from OpenStreetMap, redundant layers such as roads and water systems were removed. The data were imported into ArcGIS, followed by clipping to the study area extent and projection processing. Subsequently, the geometric area of buildings was calculated and divided by the area of the grid cell to obtain building density. Finally, the density values were assigned to the fishnet grid.
	Reconstructed Three-decade Global Fine-Grained Nighttime Light Dataset	500m	Zenodo data repository https://zenodo.org/records/15845676	After projection and clipping in ArcGIS, the processed data were assigned to the fishnet grid.
	The 30 m annual land cover datasets and its dynamics in China from 1985 to 2024	30m	Zenodo data repository https://zenodo.org/records/15853565	Land cover data for the impervious surface type were extracted in ArcGIS. The zonal statistics tool was used to summarize the impervious surface area within each fishnet cell, which was then divided by the grid cell area to calculate the impervious surface percentage. The results were finally assigned to the fishnet grid.

**Note:** Due to the large differences in the number of street-view images for July and August across different years, which resulted in insufficient data consistency, this study only collected street‑view data for June of 2014, 2017, and 2020. At the same time, to eliminate the interference of the unique meteorological conditions of the plum rain season on the analysis, meteorological factor data for the same period were incorporated into the model framework to more accurately capture the independent effects of vegetation factors on O_3_ concentration.

### 2.3. Methods

The DeepLabV3+ model was trained using the ADE20K dataset to extract the area proportions of trees, shrubs, and lawns from street-view images. In ArcGIS, the study area was divided into 1 km × 1 km fishnet grid cells. The proportions of trees, shrubs, and grass extracted from all street-view images, as well as O_3_ concentration data, meteorological data, and urban structure data, were spatially aggregated to the corresponding fishnet grid cells respectively. The average value of all samples within the same grid cell was computed as the final characteristic value for that cell. This procedure effectively reduces data redundancy and spatial dependence caused by the close street-view sampling interval, and simultaneously achieves spatial scale consistency for multi-source datasets [39,40]. After spatial alignment, the sample dataset from each grid cell was exported, and all continuous variables were normalized to eliminate the influence of scale differences on model analysis. To systematically investigate the nonlinear relationships between O_3_ concentration and the three categories of driving factors, this study employed a Bayesian XGBoost model for global importance analysis and quantification of factor contributions. Additionally, a Geographically Weighted Random Forest (GWRF) model was constructed to capture the spatial heterogeneity of the influencing mechanisms. Finally, by integrating the outputs of both models, the patterns of factor effects and regional variations were visualized multidimensionally through a combination of statistical charts and spatial distribution maps (Fig 2).

**Fig 2:**
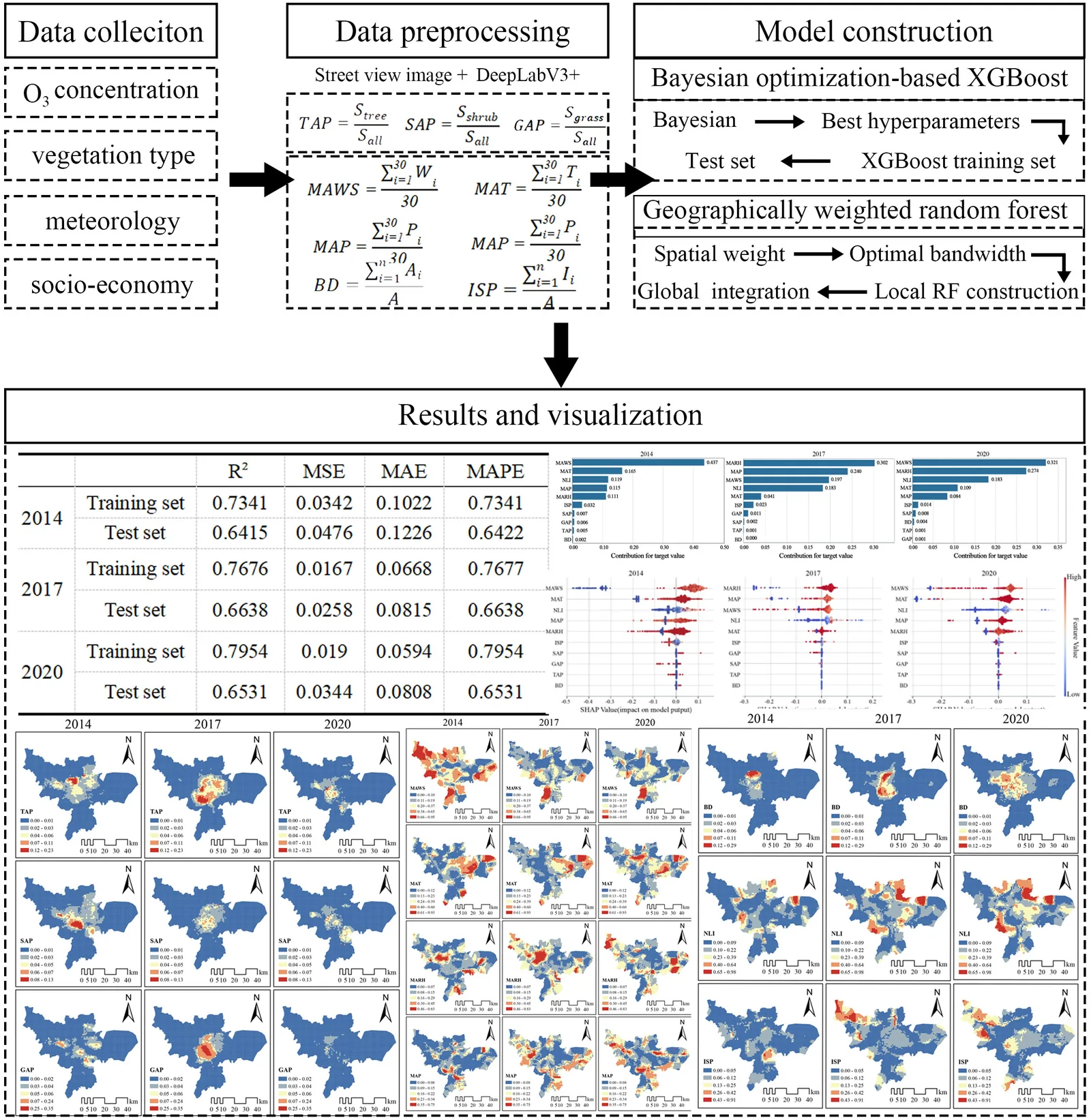
Technical roadmap. (Reference base map from the Zhejiang province map of the standard map service system, Map Approval No.: Zhejiang S (2025) No. 23).

#### 2.3.1. DeepLabV3+ semantic segmentation.

The study employed the DeepLabV3+ semantic segmentation model to extract vegetation from street-view images. The DeepLabV3+ neural network model, the latest iteration in the DeepLab series, is built on the ResNet50 backbone [41]. It innovatively integrates a decoder component into the classic DeepLabV3 architecture, combining serial and parallel structures in the Atrous Spatial Pyramid Pooling (ASPP) module and further incorporating 3 × 3 and 1 × 1 convolutional layers to form an efficient encoder-decoder framework. This design maintains computational efficiency while achieving high-precision recognition [42]. Compared to other models, DeepLabV3+ demonstrates superior performance in identifying urban street greenery structures [43].

We adopted the ADE20K dataset to train the DeepLabV3 + model. ADE20K is a semantic segmentation dataset containing over 20,000 scene-centric images. These images were sourced from public databases such as SUN and Places and are annotated with detailed pixel-level labels for objects and object parts. The dataset covers 150 semantic categories, including the three vegetation types relevant to this study: trees, shrubs, and lawns [44].

#### 2.3.2. Indicator system construction.

The study selected factors including tree area proportion (TAP), shrub area proportion (SAP), and grass area proportion (GAP) as indicators of vegetation type. Since urban O_3_ concentration levels result from the combined effects of multiple factors, meteorological and urban structural factors were also incorporated to objectively examine the role of vegetation type within the broader interactive system that includes other influencing factors [45]. Specifically, monthly average wind speed (MAWS), monthly average temperature (MAT), monthly average relative humidity (MARH), and monthly average precipitation (MAP) were selected as meteorological indicators [46]. Relevant studies indicate that urbanization and industrialization can negatively impact air quality [47]. Therefore, building density (BD), nighttime light intensity (NLI), and impervious surface proportion (ISP) were chosen as urban structural indicators (Table 2).

**Table 2:** Indicator categories and descriptions.

Indicator type	Indicator name	Calculation formula	Variable description
**Vegetation type**	TAP	$TAP=\frac{S_{tree}}{S_{all}}$	$S_{tree}$ denotes the area occupied by trees in the street-view image, and $S_{all}$ represents the total area of the street-view image.
	SAP	$SAP=\frac{S_{shrub}}{S_{all}}$	$S_{shrub}$ denotes the area occupied by shrubs in the street-view image, and $S_{all}$ represents the total area of the street-view image.
	GAP	$GAP=\frac{S_{grass}}{S_{all}}$	$S_{grass}$ denotes the area occupied by grass in the street-view image, $S_{all}$ represents the total area of the street-view image.
**Meteorology**	MAWS	$MAWS=\frac{{\sum_{i=\text{1}}^{\text{3}\text{0}}W}_{i}}{\text{3}\text{0}}$	$W_{i}$ denotes the daily average wind speed.
	MAT	$MAT=\frac{{\sum_{i=\text{1}}^{\text{3}\text{0}}T}_{i}}{\text{3}\text{0}}$	$T_{i}$ denotes the daily average surface temperature.
	MARH	$MARH=\frac{{\sum_{i=\text{1}}^{\text{3}\text{0}}R}_{i}}{\text{3}\text{0}}$	$R_{i}$ denotes the daily average relative humidity.
	MAP	$MAP=\frac{{\sum_{i=\text{1}}^{\text{3}\text{0}}P}_{i}}{\text{3}\text{0}}$	$P_{i}$ denotes the daily average precipitation.
**Urban structure**	BD	$BD=\frac{\sum_{i=\text{1}}^{n}A_{i}}{A}$	$A_{i}$ denotes the total building area within the fishnet cell, and *A* represents the area of the corresponding fishnet cell.
	NLI	–	
	ISP	$ISP=\frac{\sum_{i=\text{1}}^{n}I_{i}}{A}$	$I_{i}$ denotes the total impervious surface area within the fishnet cell, Arepresents the area of the corresponding fishnet cell.

#### 2.3.3. Bayesian optimized XGBoost model.

The Bayesian optimization algorithm was employed for hyperparameter tuning of the XGBoost model (https://doi.org/10.5281/zenodo.20008638). Bayesian Optimization, as an efficient global optimization algorithm, is widely used to determine the optimal hyperparameter values of models. Its core advantage lies in its ability to utilize prior knowledge within Gaussian Processes (GP), construct acquisition functions, dynamically update search strategies, and ultimately approximate the global optimal hyperparameter values of the objective function by analyzing the global distribution characteristics provided by the posterior distribution [48]. This algorithm typically uses GP as a probabilistic surrogate model. In the GP model, the predicted target value f(x) or any sample point x in the search space follows a normal distribution, and the probability density of the predicted target value corresponding to sample point x is P(x). The specific form of the multivariate Gaussian probability model is as follows [49]:


$$\begin{array}{c} \begin{array}{c} P(x)=\frac{\text{1}}{{\text{2}\pi }^{\frac{n}{\text{2}}}{cov}^{\frac{\text{1}}{\text{2}}}}exp\left(-\frac{\text{1}}{\text{2}}(x-\mu {)}^{T}cov(x-\mu {)}^{-\text{1}}\right) \end{array} \end{array}$$ (1)

The specific forms of the mean μ and the covariance cov are as follows:


$$\begin{array}{c} \begin{array}{c} \mu =\frac{\text{1}}{n}\sum_{i=\text{1}}^{n}x_{i} \end{array} \end{array}$$ (2)

$$\begin{array}{c} \begin{array}{c} cov=\frac{\text{1}}{n}\sum_{i=\text{1}}^{n}{\left(x_{i}-\mu \mathrm{\backslash rightleft}(x_{i}-\mu \right)}^{\text{T}} \end{array} \end{array}$$ (3)

The XGBoost model was employed to analyze the global impact of vegetation type and other factors on O_3_ concentration. Based on the gradient boosting framework, the XGBoost model incorporates several improvements over traditional gradient boosting methods, including weighted handling of data distribution, regularization to prevent overfitting, and support for parallel computing to accelerate training. It exhibits high accuracy and model robustness, performing effectively not only on large datasets but also demonstrating enhanced capability in handling relatively small datasets and preventing overfitting due to the inclusion of regularization terms in its objective function. The objective function consists of two parts: the conventional loss function

l*(φ)* and the regularization term Ω(*f*_*k*_) that controls model complexity, where *φ* denotes the parameter set:


$$\begin{array}{c} \begin{array}{c} L(\varphi )=\sum_{i=\text{1}}^{m}\text{l}\left(y_{i},\hat{y_{i}}\right)+\sum_{k=\text{1}}^{k}\Omega (f_{k}\backslash \end{array} \end{array}$$ (4)

$$\begin{array}{c} \begin{array}{c} L(\varphi )=\sum_{i=\text{1}}^{m}\left[g_{i}f_{k}\left(x_{i}\right)+\frac{\text{1}}{\text{2}}h_{i}f_{k}^{\text{2}}\left(x_{i}\right)\right]+\Omega \left(f_{k}\right) \end{array} \end{array}$$ (5)

Where, *L*(*φ*) represents the expression in linear space; *i* denotes the *i*-th sample; *k* denotes the *k*-th tree; $\hat{y_{i}}$ denotes the predicted value of the *i*-th sample *x*_*i*_; *g*_i_enotes the first-order derivative of the data point with respect to the loss function; *h*_*i*_ denotes the second-order derivative of the data point with respect to the loss function.

#### 2.3.4. SHapley Additive exPlanations model.

SHAP model was applied to interpret the prediction results of the XGBoost model and quantify the contribution of each feature to the model output. The core advantage of this method lies in its ability to evaluate the impact of individual features on the prediction outcome of a specific sample and distinguish between positive and negative effects of such impact. The formula is as follows:


$$\begin{array}{c} \begin{array}{c} y_{i}=y_{base}+f\left(X_{i_{\text{1}}}\right)+f\left(X_{i_{\text{2}}}\right)+\cdot \cdot \cdot +f\left(X_{i_{k}}\right) \end{array} \end{array}$$ (6)

Where, $f\left(X_{i_{j}}\right)$ represents the SHAP value corresponding to the feature $X_{i_{j}}$, indicating the contribution of the *j*-th feature in the *i*-th sample to the predicted value $y_{i}$.

#### 2.3.5. GWRF Model.

An improved Python version of the GWRF model [50] was introduced to uncover the local impact mechanisms of factors such as vegetation type on O_3_ concentration (https://github.com/cecil8503/GWRF). This model integrates the global learning advantages of random forests with the local adjustment characteristics of geographically weighted regression, enabling the explanation of spatial variation relationships between response and predictor variables while accounting for nonlinearities and interaction effects of the independent variables [51]. GWRF constructs a Local Random Forest (Local RF) model for each data point, focusing solely on the characteristics of neighboring data, and adjusts data sampling probabilities through a distance-weighted function [52]. Ultimately, the predictions from all local random forests are aggregated, and the final predicted value is output through a voting mechanism. The specific formula is as follows:


$$\begin{array}{c} \begin{array}{c} w_{ij}=\left\{ \begin{array}{c} {\left[\text{1}-{\left(\frac{d_{ij}}{bandwidth}\right)}^{\text{2}}\right]}^{\text{2}},d_{ij}\leq bandwidth\\ w_{ij}=\text{0},d_{ij}\leq bandwidth \end{array}\right. \end{array} \end{array}$$ (7)

Where, “bandwidth” is the distance within which observations are considered neighbors in the local computation; $d_{ij}$ is the Euclidean distance between unit *i* and unit *j*; each spatial location and its neighbors *i* (where $w_{ij}$≠0)are sequentially selected to construct the input for the Local RF. The output of the Local RF is given by the following formula:


$$\begin{array}{c} \begin{array}{c} Y_{i}=f_{\left(u_{i},v_{i}\right)}(x^{*})+\varepsilon_{i} \end{array} \end{array}$$ (8)

Where, $f_{\left(u_{i},v_{i}\right)}$ is the predicted value for spatial location *i* using the GWRF model, and $\left(u_{i},v_{i}\right)$ are the central coordinates of location *i*. During each training process of the Local RF, 70% of the data is used as the training set and 30% as the test set, with the optimal model parameters determined via a grid search optimization method.

## 3. Results

### 3.1. Spatiotemporal evolution of O_3_ concentration

By constructing a 1 km × 1 km fishnet grid for spatial assignment of O_3_ concentration, we observed significant spatiotemporal heterogeneity in O_3_ distribution (S1, S2 and S3 Tables in S1 File). Overall, O_3_ concentrations showed a decreasing trend. The area with early-summer O_3_ concentrations in the range of 120.01–160.00 μg/m^3^ decreased from 2,100 km^2^ in 2014–189 km^2^ in 2017, and further dropped to 0 km^2^ by 2020. Concurrently, the area with moderate O_3_ concentrations (100.01–120.00 μg/m^3^) increased substantially, rising from 740 km^2^ to 2,302 km^2^ (Table 3). Spatially, the pattern generally exhibited “higher in the central areas and lower in the periphery.” Local O_3_ concentrations in the northwestern core urban areas—such as northwestern Qiantang District, northern Linping District, and central Xihu District—were significantly higher than surrounding regions. In addition, most parts of Xiaoshan District in the southeast, except its northern portion, also showed relatively high O_3_ concentrations (Fig 3).

**Table 3:** Tiered area coverage of O_3_ concentration from 2014 to 2020 Unit: km^2^.

O_3_ concentration level	Area in 2014	Area in 2017	Area in 2020
**0.00-60.00 (Extremely low)**	761	489	489
**60.01-100.00 (Relatively low)**	27	841	837
**100.01-120.00 (Moderate)**	740	2,109	2,302
**120.01-160.00 (High)**	2,100	189	0
**160.01-200.00 (Extremely high)**	0	0	0

**Fig 3:**
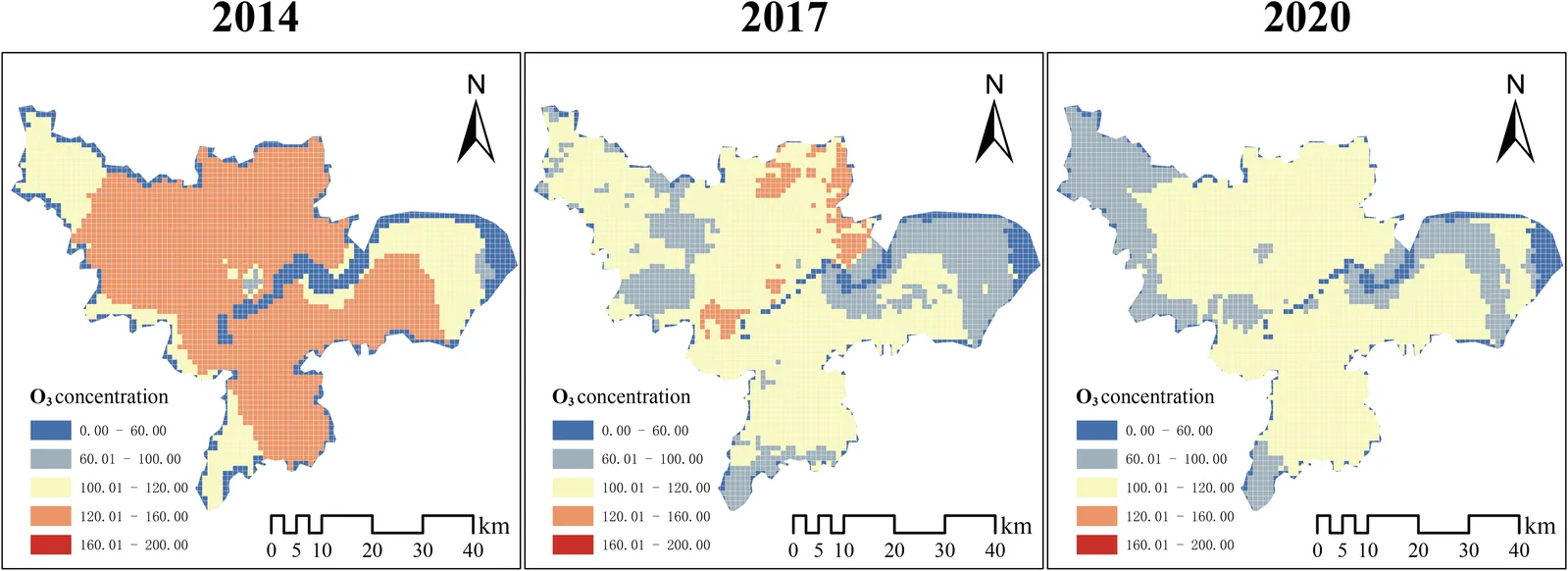
Spatiotemporal evolution of O_3_ concentration. (Reference base map from the Zhejiang province map of the standard map service system, Map Approval No.: Zhejiang S (2025) No. 23).

### 3.2. Global mechanisms influencing O_3_ concentration

Using the O_3_ concentration data and the three categories of factor data as inputs (S4, S5 and S6 Tables in S1 File), the Bayesian optimized XGBoost model demonstrated good predictive performance in all three years (2014, 2017, and 2020). On the training set, the R^2^ ranged from 0.7341 to 0.7954, MSE from 0.0167 to 0.0342, RMSE from 0.1291 to 0.1850, and MAE from 0.0594 to 0.1022. On the test set, the R^2^ ranged from 0.6415 to 0.6638, MSE from 0.0258 to 0.0476, RMSE from 0.1607 to 0.2182, and MAE from 0.0808 to 0.1226 (Table 4). Overall, the model achieved good fitting performance on the training set and stable generalization ability on the test set, with prediction errors remaining at a low level overall and no obvious overfitting, indicating that the optimized XGBoost model has reliable predictive performance.

**Table 4:** Regression performance metrics for the Bayesian-optimized XGBoost model.

Year	Dataset	R²	MSE	RMSE	MAE
**2014**	Training set	0.7341	0.0342	0.1850	0.1022
	Test set	0.6415	0.0476	0.2182	0.1226
**2017**	Training set	0.7676	0.0167	0.1291	0.0668
	Test set	0.6638	0.0258	0.1607	0.0815
**2020**	Training set	0.7954	0.0190	0.1379	0.0594
	Test set	0.6531	0.0344	0.1856	0.0808

Compared to meteorological and urban structural factors, vegetation-type factors contributed the least to O_3_, with importance scores ranging only from 0.001 to 0.011 during 2014−2020. Among them, SAP and GAP showed relatively higher average contributions (0.006), while TAP had the lowest average contribution (0.002). In terms of trends, the contributions of both TAP and GAP generally decreased over time: TAP declined from 0.005 in 2014 to 0.001 in 2020, and GAP first rose from 0.006 in 2014 to 0.011 in 2017, then fell back to 0.001 in 2020, representing the largest decrease (−83.33%). In contrast, the contribution of SAP overall increased, dropping from 0.007 to 0.002 and then rising again to 0.008. Meteorological factors exhibited the highest importance, especially MAWS, with contributions of 0.437, 0.197, and 0.321 in 2014, 2017, and 2020, respectively, ranking first in both 2014 and 2020. Urban structural factors showed intermediate importance, with NLI having the highest contribution—even exceeding those of meteorological factors such as MAP, MARH, and MAT—reaching 0.119, 0.183, and 0.183 from 2014 to 2020. However, the importance of BD in 2014 and 2017 was lower than that of the three vegetation-type factors, at only 0.002 and 0.000, respectively (Fig 4).

**Fig 4:**
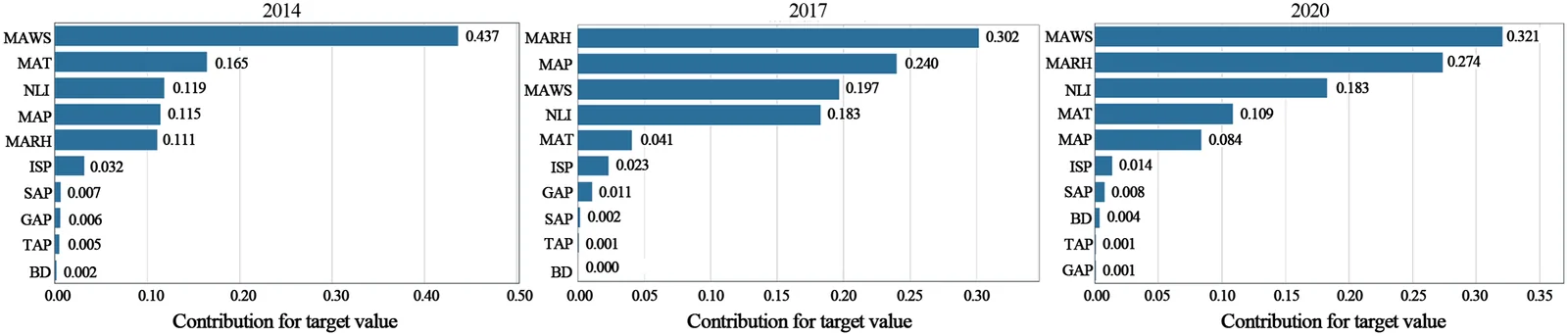
SHAP feature contribution plot.

In terms of influence trends, the red sample points of SAP are predominantly distributed in the positive region of the SHAP values, indicating that SAP has a positive effect on O_3_ concentration. The red sample points of TAP are distributed across both positive and negative regions of the SHAP values, suggesting that TAP has context-dependent effects, which may manifest mainly as negative effects in some areas and as positive effects in others. The distribution of red sample points for GAP is similar to that of TAP, with a greater concentration in the negative region, reflecting a certain degree of negative influence of GAP on O_3_. This negative trend was most pronounced in 2017 (Fig 5).

**Fig 5:**
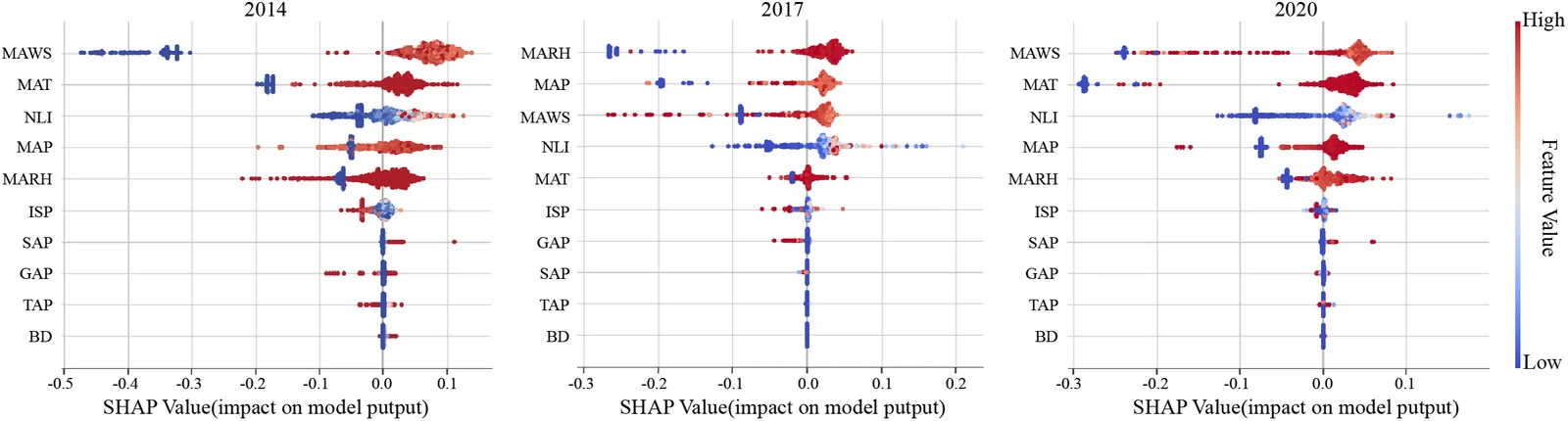
SHAP summary plot.

The permutation test shows that the proportions of positive SHAP values in the high-value areas of TAP and SAP, as well as the proportion of negative SHAP values in the high‑value area of GAP, are significantly higher than the 95th percentile of the null distribution (p < 0.01). Therefore, despite the small absolute SHAP values, their positive/negative directions are statistically significant and not merely random noise.

### 3.3. Local mechanisms influencing O_3_ concentration

#### 3.3.1. Comprehensive characteristics of local importance of influencing factors.

Using the datasets and their corresponding coordinates as inputs (S7, S8, and S9 Tables in S1 File), the GWRF results indicate that meteorological factors overall exhibited the highest mean local importance (S10, S11 and S12 Tables in S1 File). Among these, the mean importance of MAWS showed a significant decreasing trend (from 0.3690 in 2014 to 0.1679 in 2020), while the mean importance of the remaining factors slightly increased. Urban structural factors displayed a higher mean importance than vegetation-type factors overall, with an upward trend. Within this category, the mean importance of BD in 2014 and 2017 was notably lower than that of the vegetation-type factors. The mean importance of vegetation-type factors showed a marked decreasing trend. By 2020, the mean importance values of TAP, SAP, and GAP had decreased to 0.0037, 0.0038, and 0.0026, respectively, all lower than the mean importance of BD (0.0097) (Table 5).

**Table 5:** Mean local importance of factors.

Influencing factors	Year		
	2014	2017	2020
**TAP**	0.0072	0.0097	0.0037
**SAP**	0.0072	0.0041	0.0038
**GAP**	0.0076	0.0132	0.0026
**MAWS**	0.3690	0.1759	0.1679
**MAT**	0.2195	0.2034	0.2229
**MARH**	0.1326	0.1694	0.1643
**MAP**	0.0911	0.1395	0.1384
**BD**	0.0046	0.0064	0.0097
**NLI**	0.1170	0.1899	0.1944
**ISP**	0.0442	0.0887	0.0922

#### 3.3.2. Local effects of vegetation-type Factors on O_3_ concentration.

The spatial distribution of vegetation types exhibits significant spatial clustering effects. The proportions of each vegetation type generally follow a pattern of higher values in the central areas and lower values in the surrounding regions. Areas with higher vegetation proportions are primarily located in central districts such as Gongshu, Shangcheng, Binjiang, and Xihu.

Specifically, regions where the importance of TAP and SAP exceeded 0.01, and those where GAP exceeded 0.02, all showed a shrinking spatial trend. The spatial location of extremely high TAP importance shifted from northern Xihu District and Gongshu District in 2014 to southern Xihu District and Binjiang District in 2017. Additionally, both the higher and extremely high importance areas of TAP displayed a pattern of initial increase followed by decrease (the area of higher importance increased from 26 km^2^ in 2014–165 km^2^ in 2017, then decreased to 21 km^2^ in 2020; the area of extremely high importance rose from 27 km^2^ in 2014–46 km^2^ in 2017, and further declined to 3 km^2^ in 2020). In 2014, higher and extremely high importance areas of SAP were observed in southern Xihu District and western Xiaoshan District, which gradually diminished thereafter (the area of higher importance decreased from 56 km^2^ to 14 km^2^; the area of extremely high importance decreased from 52 km^2^ to 1 km^2^). The higher and extremely high importance areas of GAP were notably clustered only in 2017 (higher importance area: 157 km^2^; extremely high importance area: 48 km^2^), distributed across eastern Xihu District, southern Gongshu District, Binjiang District, and Shangcheng District (Fig 6 and Table 6).

**Table 6:** Area distribution of vegetation-type factors by importance level.

Factors	Level	Value range	2014	2017	2020
**TAP**	Extremely low	0.00-0.01	293,8	300,0	323,6
	Relatively low	0.02-0.03	418	191	268
	Moderate	0.04-0.06	219	226	100
	Relatively high	0.07-0.11	26	165	21
	Extremely high	0.12-0.23	27	46	3
**SAP**	Extremely low	0.00-0.01	284,9	315,7	319,5
	Relatively low	0.02-0.03	504	320	283
	Moderate	0.04-0.05	167	127	135
	Relatively high	0.06-0.07	56	24	14
	Extremely high	0.08-0.13	52	0	1
**GAP**	Extremely low	0.00-0.02	309,6	319,2	352,1
	Relatively low	0.03-0.04	372	172	99
	Moderate	0.05-0.06	122	59	7
	Relatively high	0.07-0.24	38	157	1
	Extremely high	0.25-0.35	0	48	0

**Fig 6:**
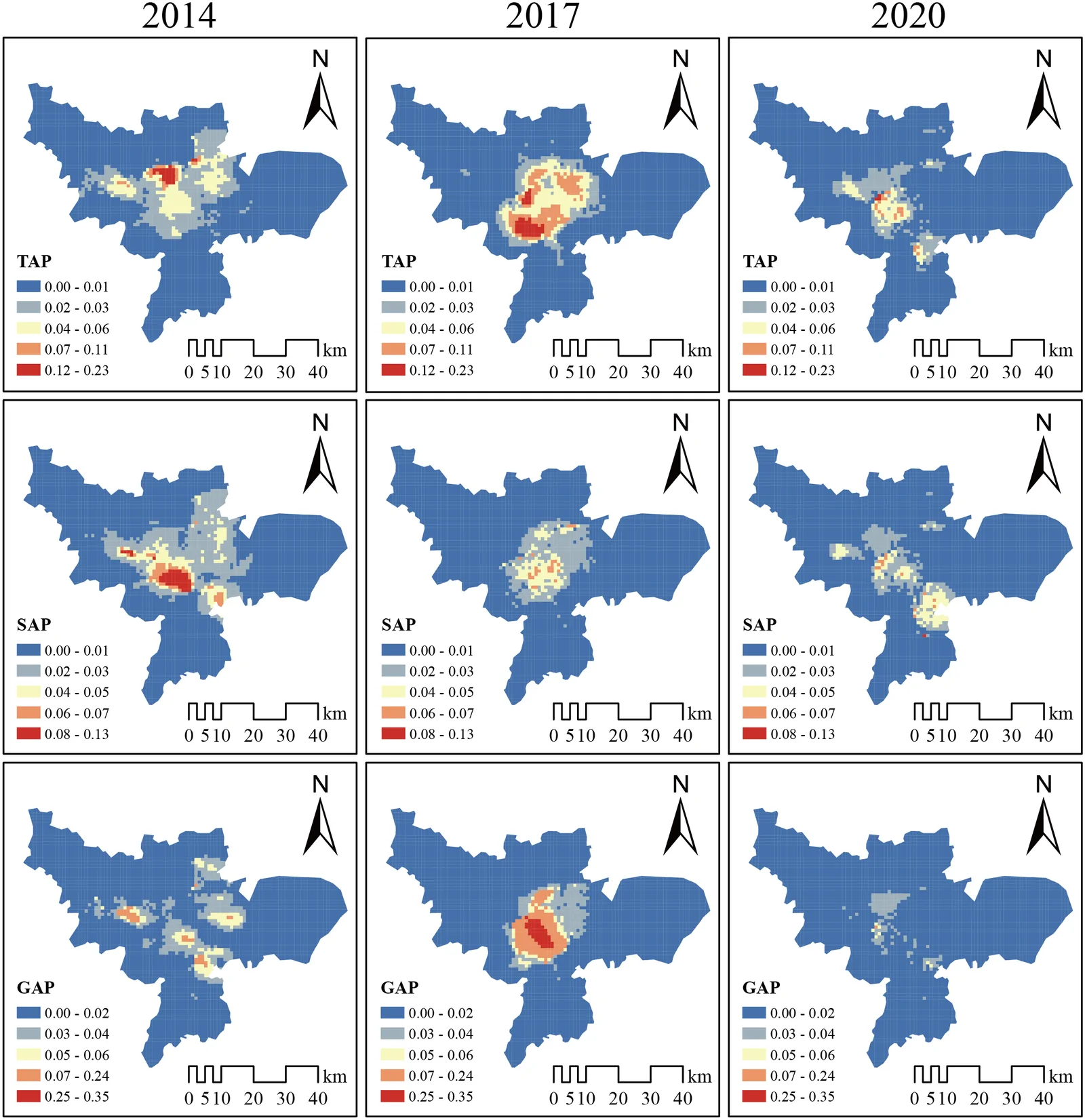
Local importance of vegetation-type factors. (Reference base map from the Zhejiang province map of the standard map service system, Map Approval No.: Zhejiang S (2025) No. 23).

#### 3.3.3. Local effects of meteorological factors on O_3_ concentration.

Overall, areas with high values of meteorological factors are mainly distributed on the periphery of the central urban area, including Yuhang District, Xihu District, Xiaoshan District, and others.

Specifically, MAWS and MAT exhibited a decreasing trend, while MARH and MAP showed an increasing trend. In 2014, areas with relatively high and extremely high MAWS values were distributed across the entire Yuhang District, western Linping District, southern and eastern Xiaoshan District. Subsequently, their spatial extent gradually contracted (the area of relatively high importance decreased from 1,116 km^2^ to 207 km^2^, and the area of extremely high importance decreased from 586 km^2^ to 102 km^2^). By 2020, large clustered areas of high MAWS values remained only in southeastern Xiaoshan District. Areas with relatively high and extremely high MAP values were mainly concentrated in Yuhang District and Xiaoshan District, with their extent gradually shrinking (the area of relatively high importance decreased from 440 km^2^ to 385 km^2^, and the area of extremely high importance decreased from 241 km^2^ to 171 km^2^). Areas with relatively high and extremely high MARH values were primarily distributed in northwestern regions such as Yuhang District, Xihu District, and Gongshu District, with additional clusters in the northeast (the area of relatively high importance increased from 191 km^2^ to 287 km^2^, and the area of extremely high importance increased from 188 km^2^ to 248 km^2^). The high-value areas in the northwest first expanded and then contracted, while a new high-value center gradually developed further northwest of this region. Areas with relatively high and extremely high MAP values initially appeared in southern Xihu District and southern Xiaoshan District, then gradually expanded westward and eastward, eventually extending to southern Yuhang District, northern Xiaoshan District, and southern Qiantang District (the area of relatively high importance increased from 138 km^2^ to 580 km^2^, and the area of extremely high importance increased from 75 km^2^ to 129 km^2^) (Fig 7 and Table 7).

**Table 7:** Area distribution of meteorological factors by importance level.

Factors	Level	Value range	2014	2017	2020
**MAWS**	Extremely low	0.00-0.10	622	129,4	142,7
	Relatively low	0.11-0.19	495	1285	1123
	Moderate	0.20-0.37	809	682	769
	Relatively high	0.38-0.65	111,6	248	207
	Extremely high	0.66-0.95	586	119	102
**MAT**	Extremely low	0.01-0.12	157,6	133,1	110,0
	Relatively low	0.13-0.23	812	120,9	122,7
	Moderate	0.24-0.39	559	601	745
	Relatively high	0.40-0.60	440	339	385
	Extremely high	0.61-0.93	241	148	171
**MARH**	Extremely low	0.00-0.07	139,7	896	103,6
	Relatively low	0.08-0.15	121,4	116,6	106,9
	Moderate	0.16-0.29	638	105,6	988
	Relatively high	0.30-0.45	191	274	287
	Extremely high	0.46-0.83	188	236	248
**MAP**	Extremely low	0.00-0.08	208,5	117,9	122,2
	Relatively low	0.09-0.15	974	953	102,2
	Moderate	0.16-0.22	356	795	675
	Relatively high	0.23-0.34	138	613	580
	Extremely high	0.35-0.75	75	88	129

**Fig 7:**
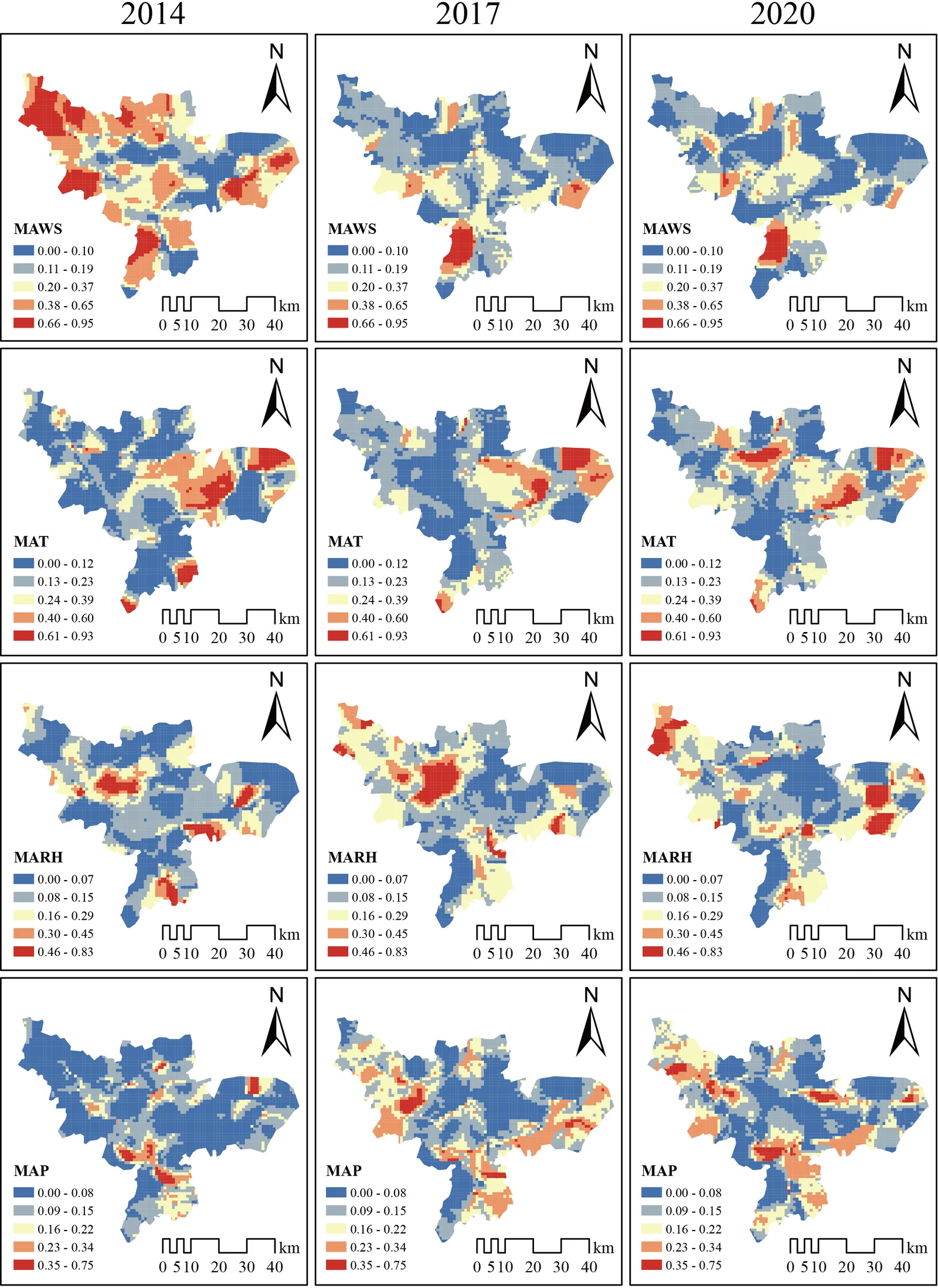
Local importance of meteorological factors. (Reference base map from the Zhejiang province map of the standard map service system, Map Approval No.: Zhejiang S (2025) No. 23).

#### 3.3.4. Local effects of urban structural factors on O_3_ concentration.

Overall, the distribution of urban structural factors shows significant spatial heterogeneity. BD is mainly concentrated in the central areas, while NLI and ISP are predominantly distributed on the periphery of the central urban zone. Specifically, BD, NLI, and ISP all exhibited an increasing trend. In 2014, areas with relatively high and extremely high BD values were primarily located in northern Xihu District and Gongshu District, subsequently expanding southward to Shangcheng District and Binjiang District. However, by 2020, the extent of extremely high BD areas had decreased (from 33 km^2^ in 2014–44 km^2^ in 2017, then down to 8 km^2^ in 2020), and high-value areas became more spatially fragmented. NLI displayed a marked trend of expansion from the periphery toward the interior (the area of relatively high importance increased from 160 km^2^ to 373 km^2^, and the area of extremely high importance rose from 39 km^2^ to 210 km^2^). High-value NLI areas were mainly clustered in southern Xihu District, western Qiantang District, eastern Yuhang District, and Linping District. ISP initially showed relatively high and extremely high importance in the southeastern region (central Xiaoshan District) in 2014, then gradually shifted to northwestern Yuhang District. The area of extremely high ISP importance first increased and then decreased (from 6 km^2^ in 2014–104 km^2^ in 2017, then down to 56 km^2^ in 2020) (Fig 8 and Table 8).

**Table 8:** Area distribution of urban structural factors by importance level.

Factors	Level	Value range	2014	2017	2020
**BD**	Extremely low	0.00-0.01	325,8	322,8	279,4
	Relatively low	0.02-0.03	234	149	362
	Moderate	0.04-0.06	74	123	324
	Relatively high	0.07-0.11	29	84	140
	Extremely high	0.12-0.29	33	44	8
**NLI**	Extremely low	0.00-0.09	212,8	151,1	149,2
	Relatively low	0.10-0.22	915	107,4	104,5
	Moderate	0.23-0.39	386	454	508
	Relatively high	0.40-0.64	160	427	373
	Extremely high	0.65-0.98	39	162	210
**ISP**	Extremely low	0.00-0.05	258,5	178,9	172,8
	Relatively low	0.06-0.12	875	112,3	985
	Moderate	0.13-0.25	108	378	608
	Relatively high	0.26-0.42	54	234	251
	Extremely high	0.43-0.91	6	104	56

**Fig 8:**
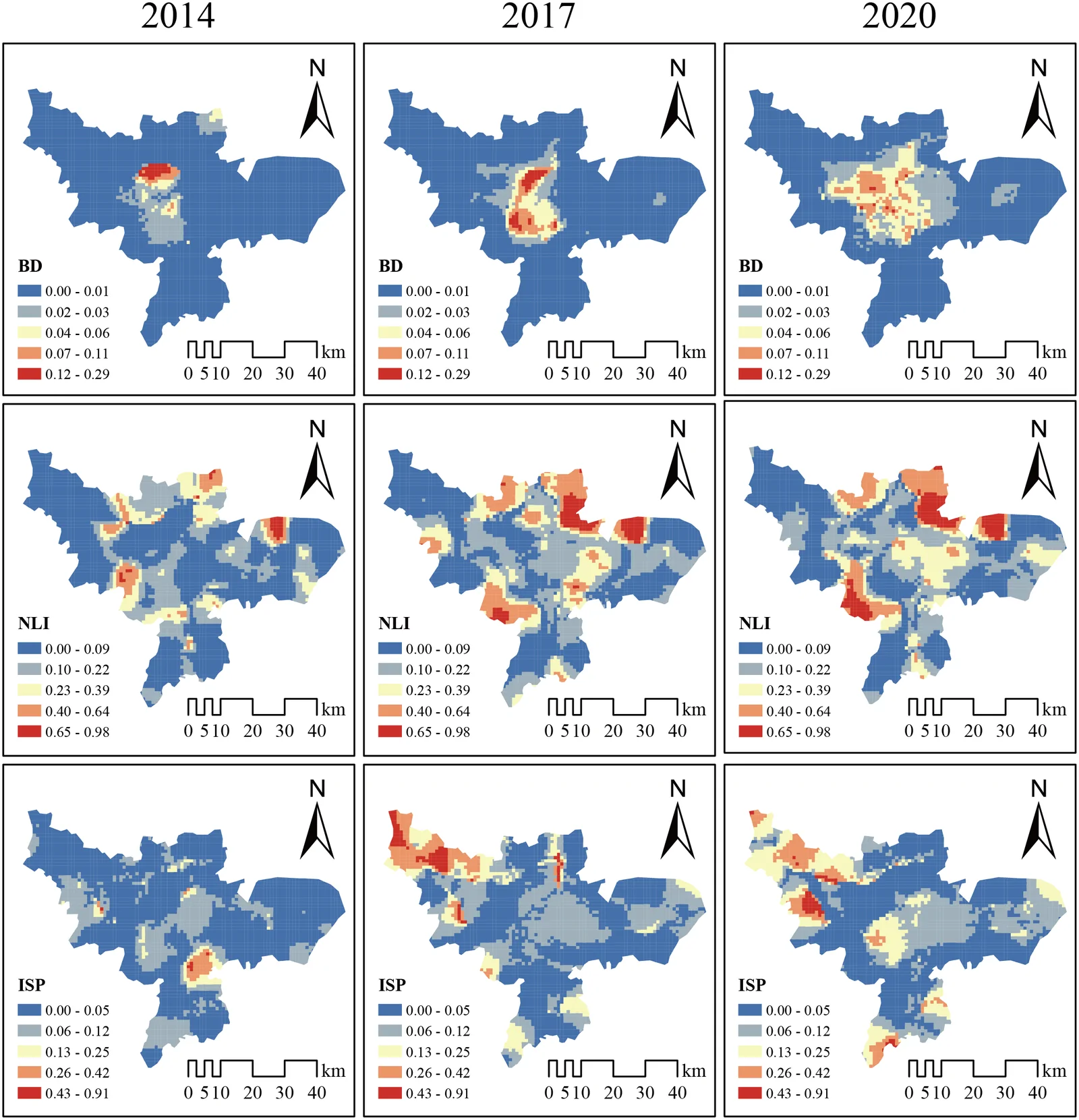
Local importance of urban structural factors. (Reference base map from the Zhejiang province map of the standard map service system, Map Approval No.: Zhejiang S (2025) No. 23).

## 4. Discussion

### 4.1. Spatiotemporal distribution characteristics of O_3_ concentration

O_3_ pollution was relatively severe around 2014, after which it showed significant improvement. This aligns with findings from studies such as [53], which indicate that volatile organic compounds (VOCs) emissions during the warm season are a major cause of elevated O_3_ concentrations. Additionally, higher temperatures in 2014 enhanced photochemical reaction rates, often accompanied by sunny, dry, and stagnant weather conditions, leading to the accumulation of O_3_ [54]. In 2016, the G20 Summit was held in Hangzhou. To ensure air quality met standards during the conference, the Zhejiang Provincial Government implemented a series of coordinated air quality control measures at the district level, reducing emissions from industrial, transportation, residential, and power sectors [55], which effectively alleviated O_3_ pollution. In recent years, the government has promoted the “Blue Sky Protection Campaign”, which has reduced emissions of nitrogen oxides (NO_x_)—a key precursor to O_3_—contributing to the continuous decline in O_3_ concentrations [56]. Xihu District and other core urban areas, as well as Xiaoshan District, have been severely polluted. The primary reason lies in Xiaoshan District, a major industrial agglomeration zone with significant VOCs emissions [57]. Core urban areas, due to high population density and substantial traffic-related NO_x_ emissions, generate O_3_ through photolysis under sunlight [58]. It is noteworthy that O_3_ concentration is influenced by the NO_x_/VOCs emission ratio. When the NO_x_/VOCs ratio in core urban areas is below 8, ozone formation is under VOCs-limited conditions, and reducing NO_x_ emissions through traffic control measures may paradoxically increase O_3_ concentrations [59].

### 4.2. Impact of vegetation types on O_3_ concentration

This study utilized semantic segmentation of street-view imagery to obtain the proportion of trees, shrubs, and grass, revealing the complex mechanisms by which micro-scale vegetation types influence O_3_ concentration from a human perspective. By accurately representing the green space structure as perceived in the human field of view, this approach addresses the gap in prior research that predominantly focused on macro-scale green space characteristics.

At the global scale, the contribution of vegetation-type factors to O_3_ concentration was lower than that of meteorological and urban structural factors. However, in 2017, the contribution of GAP was relatively high, reaching 0.011. In 2017, the three vegetation types showed no significant impact on O_3_ concentration, aligning with the experimental results of [60] across different plant communities. SAP tends to have a positive influence when its proportion is relatively high, which is related to the intensive application of shrubs in Hangzhou’s green spaces and the selection of specific tree and shrub species(e.g., Euonymus japonicus ‘Aureo-marginatus’, Prunus cerasifera ‘Pissardii’) [61,62]. Due to the high planting density of shrubs, the BVOCs emitted by densely planted vegetation typically exceed the amount taken up through stomata, thereby leading to an increase in O_3_ concentration [63]. GAP showed a significantly negative effect on O_3_ concentration when present in higher proportions. This may be because, compared to trees and shrubs, grass has a smaller biomass and limited BVOCs emissions, resulting in a net absorption effect on O_3_. Notably, the average contribution of TAP was 0.002, significantly lower than that of SAP and GAP, and it exhibited a slight positive influence or negative influence. This aligns with the findings of [64], which indicate that broadleaf forests emit approximately six times more BVOCs than grassland vegetation. In the dense urban blocks of the central city, influenced by high NO_x_ concentrations and a strong urban heat island effect, the BVOCs emission effect is amplified. Meanwhile, the dry deposition uptake effect of trees on O_3_ concentration is comparable to their O_3_ formation effect, which may lead to a slight increase in O_3_ concentration [65,66]. Consequently, the O_3_-reducing effect of trees is substantially offset, resulting in a relatively weak net impact of trees on O_3_ concentration, characterized by strong variability and spatially alternating positive and negative effects. This variability makes it difficult for models to capture stable relationships, ultimately leading to a lower contribution compared to shrubs and grass.

At the local scale, the influence of vegetation types on O_3_ concentration showed significant spatiotemporal heterogeneity. The importance of vegetation-type factors was markedly higher in areas with severe pollution. In the high O_3_ pollution areas of 2014, TAP had higher importance in northern Xihu District and Gongshu District, while SAP was more important in eastern Xihu District and western Binjiang District. Northern Xihu District and Gongshu District are early urbanized core areas of Hangzhou with high vehicle ownership and substantial emissions of nitrogen oxides (NO_x_) and VOCs. Meanwhile, the greening pattern in this area is dominated by planted tree forests (including Platanus × acerifolia, Salix babylonica, Cinnamomum camphora, etc.), which exhibit high BVOC emissions [62]. Broadleaf tree species also reduce wind speed and weaken air circulation, thereby hindering the dispersion of O_3_ and other pollutants and causing their accumulation [67], which in turn increases O_3_ concentration. The eastern part of Xihu District and the western part of Binjiang District belong to the core scenic area of West Lake Scenic Area, characterized by a high proportion of shrubs and dense vegetation [68]. Flower borders and sparse woodland grasslands are planted with abundant shrub communities [69]. To the west lies the central urban area, with the transitional zone dominated by scattered street trees [70]. NO_x_ emissions from traffic in the urban area can easily disperse to this area, intensifying photochemical reactions [70]. Consequently, the importance of SAP is relatively high in eastern Xihu District and western Binjiang District.In 2017, areas with higher and extremely high TAP importance shifted to southern Xihu District and Binjiang District. Hangzhou has gradually transitioned from a development model centered on West Lake to a new era spanning the Qiantang River, with Binjiang District emerging as a hub for high-tech industries [71]. As a newly developed urban district, industrial and traffic emissions have increased [72], maintaining a relatively high level of NO_x_ concentration in the region. Against this background, the photochemical reactions between BVOCs emitted by trees and anthropogenic NO_x_ are intensified, causing TAP to exacerbate O_3_ pollution [73]. Southern Xihu District serves as a key transportation node to the West Lake Scenic Area, with well-developed tourism infrastructure and significant emissions from traffic and catering [74], contributing to increased O_3_. Meanwhile, government-led urban greening projects [75] have increased tree coverage, potentially enhancing net O_3_ formation through BVOCs emissions. The areas with higher and extremely high GAP importance were most extensive in 2017, covering much of the central urban core, mainly distributed in southeastern Xihu District, Binjiang District, southern Gongshu District, and Shangcheng District. This pattern aligns with the fragmented, small-scale green space structure typical of high-density built-up areas, where grass becomes the dominant green space type. The area of higher and extremely high GAP importance showed an initial increase followed by a decrease, with significant fluctuations (an increase of 439%, followed by a decrease of 99.51%). This may indicate the short-term efficiency of grass in regulating O_3_ concentration. Furthermore, urban expansion in Hangzhou has led to increased fragmentation of green spaces [76]. When the patch size of green spaces decreases below a certain threshold, the negative regulatory effect of grass on O_3_ concentration weakens or even disappears [6].

It is worth noting that TAP and GAP both exhibit large areas of importance overlap with BD. High-density built-up areas such as Gongshu District, Shangcheng District, and Xihu District have high BD, and these areas possess abundant green spaces, well-planned parks, and riverside landscapes, with good greening levels and numerous planted trees [77]. The combination of high tree density and compact building spaces can significantly reduce wind speed, thereby greatly weakening the ventilation capacity of street canyons. Trees may also affect turbulent energy distribution and suppress large‑scale eddies, hindering the outward transport of pollutants [78,79]. This may lead to TAP exacerbating O_3_ accumulation in high-BD areas. However, a study by [78] found that even within the same street environment, factors such as tree height, tree morphology, and crown diameter cause significant variations in pollutant concentrations, indicating that the aerodynamic effects associated with TAP still have an impact on O_3_ concentration. In a simulation study by [80], when the street aspect ratio was high, air pollutant concentration did not increase with increasing leaf area density; instead, pollution was most severe at medium density. This suggests a non-linear relationship between vegetation density and air pollutant concentration. Under high-BD conditions, the O_3_ ventilation efficiency of sparse vegetation is higher than that of a tree-free scenario, indicating that sparse vegetation independently improves O_3_ dispersion, rather than relying solely on the “trapping” effect of high-BD streets. Grass is low-lying, has a small frontal area, and offers little resistance to airflow; therefore, it does not alter the macroscopic wind field structure and has relatively small aerodynamic effects on O_3_ concentration [18]. In areas with high BD, GAP tends to have a stronger effect on absorbing and depositing high O_3_ concentrations [81], and it can also indirectly reduce O_3_ concentration by lowering temperature through transpiration, thereby inhibiting photochemical reactions [82].

Integrating global and local scale analyses, TAP, SAP, and GAP exhibit significantly differentiated regulatory effects on near-surface O_3_ concentration. Trees have a strong BVOCs emission capacity; the overall net effect of TAP on O_3_ reduction is weak and shows spatial positive-negative fluctuations, primarily manifesting as a positive effect in urban areas such as Xihu District and Binjiang District. Shrubs account for a relatively large proportion in urban green spaces such as the West Lake Scenic Area, and some species (e.g., Euonymus japonicus ‘Aureo-marginatus’, Prunus cerasifera ‘Pissardii’) have strong BVOCs emission capacity; SAP generally exhibits a positive promoting effect on O_3_ concentration. Grass is more important in core urban areas; due to its small biomass and low BVOC emission level, GAP mainly plays a role in absorbing and reducing O_3_ concentration.

### 4.3. Synergistic effects of vegetation types with other factors

In areas with high meteorological factor importance, the importance of vegetation factors is relatively low. These regions are mainly located in the peripheral ecological zones or outer suburban areas of the city, including western Xihu District, western and northern Yuhang District, and southern Xiaoshan District. Due to lower human activity intensity in these peripheral areas, meteorological conditions become the primary factors influencing O_3_ generation, chemical transformation, transport, and removal. For example, higher humidity and lower temperatures are unfavorable for the formation and accumulation of O_3_. This aligns with the findings of [83] regarding O_3_ concentrations in urban and suburban areas of Beijing. Influenced by the early-summer southeastern monsoon, ozone tends to be transported southward during high-ozone pollution days in coastal regions such as Jiangsu [84], which may contribute to increased O_3_ concentrations in the urban periphery. Under the dominant influence of meteorological conditions, the regulatory role of vegetation is considerably limited.

Areas with high BD importance also exhibited high importance for trees and grass. Regions where high BD importance overlapped with high TAP and SAP importance were primarily concentrated in northern Xihu District and southwestern Gongshu District in 2014, subsequently expanding southeastward to southeastern Xihu District and northern Binjiang District. Northern Xihu District and southwestern Gongshu District encompass the city center around Wulin Square, surrounded by traditional commercial zones and tourist facilities with high population density [85]. In the early stages of urban development, extensive tree planting along urban streets and the addition of public green spaces such as Xixi Wetland Park were implemented [86]. Later, government-led urban expansion along the Qiantang River promoted development in areas like northern Binjiang District, a hub for high-tech industries [87], characterized by compact development patterns [88,89]. Simultaneously, green space ratios increased in newly developed residential and commercial areas [90]. Against this backdrop, the tree-grass planting model has emerged as a strategy to accommodate intensive land use while balancing ecosystem service functions.

Areas with high ISP importance also showed high importance for SAP and GAP. The overlapping regions of high importance were primarily located in central Xiaoshan District in 2014 and gradually diminished thereafter. This region is dominated by industrial clusters with sparse vegetation, where only scattered small-scale green spaces consisting mainly of shrubs and grass are distributed [91]. Subsequently, further expansion of industrial land has encroached upon green spaces [92].

### 4.4. Strategies and recommendations

#### 4.4.1. Precise zoning control for O_3_ pollution.

In response to the spatial heterogeneity of O_3_ concentrations, a zoning control strategy of “strict control in the northwest and optimization in the southeast” should be implemented. In the northwestern core urban areas (Qiantang District, Linping District, and Xihu District), priority should be given to reducing O_3_ precursor emissions, strengthening in-depth treatment of VOCs from industrial enterprises, and enhancing control over vehicle exhaust emissions. Simultaneously, the layout of urban ventilation corridors should be optimized by reducing building density and increasing open spaces to improve pollutant dispersion capacity. In Xiaoshan District, efforts should focus on controlling industrial emission sources, combined with optimized vegetation configuration, to establish an O_3_ concentration regulation system integrating “pollution source control and vegetation adjustment.”

#### 4.4.2. Optimizing green space vegetation configuration patterns.

Based on the O_3_ regulation effects of different vegetation types, an urban green space configuration model of “trees as the main component, shrubs as supplements, and grass as accents” should be established. In areas with high potential O_3_ risks (such as industrial clusters and along major traffic corridors), priority should be given to planting tree species with low BVOCs emissions and strong adsorption capacity (e.g., camphor, osmanthus, etc.), complemented by a moderate number of shrubs to form composite forest belts, thereby enhancing the adsorption and purification efficiency for O_3_ and its precursors. In land-constrained areas such as industrial zones, commercial districts, and residential communities, the continued expansion of small-scale green spaces like lawns should be promoted to leverage their short-term mitigating effect on O_3_ pollution.

To address the fragmentation of vegetation coverage observed from a street-view perspective, the optimization of green space through “corridor connectivity and patch integration” should be advanced. By leveraging linear urban spaces such as roads and rivers, continuous vegetation corridors should be established to improve the connectivity of green spaces. In densely built areas, three-dimensional greening methods such as rooftop greening and vertical greening should be adopted to supplement vegetation coverage, reduce the proportion of impervious surfaces, improve the local microclimate, and enhance the regulatory capacity for O_3_.

#### 4.4.3. Strengthening multi-factor collaborative control.

Given the dominant role of meteorological and urban structural factors in influencing O_3_ concentrations, a collaborative control mechanism integrating “meteorological early warning—source emission regulation—vegetation adjustment” should be established. During early summer, dry spells embedded in the Meiyu season are accompanied by short-term favorable meteorological anomalies for ozone formation, including enhanced solar radiation, elevated temperature, and reduced humidity, which consequently lead to the accumulation and excessive concentration of O_3_. Therefore, targeted meteorological early warnings should be issued in advance, and differentiated emission reduction measures for industrial and transportation sources should be implemented simultaneously. Considering the localized influence characteristics of urban structural factors, the control of nighttime VOCs emissions should be strengthened in areas with high building density and strong nighttime light intensity to reduce the formation of O_3_ precursors, thereby fundamentally curbing O_3_ production during Meiyu dry spells. Furthermore, persistent cloudy, rainy, low-light, and humid conditions during the early summer Meiyu season can weaken vegetation physiological activity and purification capacity. Thus, refined vegetation management and maintenance should be enhanced to stabilize and sustain the regulatory and purification functions of vegetation for O_3_.

### 4.5. Research limitations and future prospects

Although this study reveals the mechanisms by which vegetation types influence O_3_ concentrations from a street-view perspective, several limitations remain: First, it did not explore the synergistic effects of different combinations of trees, shrubs, and grass on O_3_ concentrations. Existing studies have focused primarily on the independent effects of individual vegetation types, overlooking the roles of proportional allocation and local spatial arrangements of different vegetation types in regulating O_3_. Future research could conduct controlled experiments with various green space combination patterns, establishing experimental groups with different vegetation proportions and spatial layouts to quantify their synergistic effects on O_3_ concentrations and propose optimal green space configuration schemes. Second, the BVOCs emission characteristics of vegetation types were not incorporated into the analysis. Different tree species or plant varieties may exhibit significant variations in BVOCs emission intensity, potentially leading to distinct impacts on O_3_ concentrations. This study indirectly reflected such influences through vegetation proportions without delving into interspecific differences among specific plant species.

Third, this study does not consider the seasonal dynamics of vegetation or intra-seasonal monthly variations. Vegetation has higher coverage and stronger physiological activity in summer, and its regulatory effect on O_3_ may differ from those in other seasons. Meanwhile, influenced by meteorological conditions and phenological rhythms, summer O_3_ concentrations also show distinct monthly variations. Future research could incorporate observed vegetation BVOC emission data to construct a coupling framework of “vegetation proportion — BVOC emissions — O_3_ concentration”. It is also suggested to expand the study period to include spring, autumn, and winter, and further analyze the regulatory differences of vegetation types on O_3_ concentrations within the same season, so as to systematically reveal the seasonal evolution and monthly variation characteristics of O_3_ pollution.

## 5. Conclusion

This study focused on eight main urban districts of Hangzhou. Based on early-summer O_3_ concentration data, street-view imagery, meteorological data, and urban structural data from 2014 to 2020, it employed DeepLabV3 + semantic segmentation, Bayesian-optimized XGBoost, SHAP interpretation, and GWRF models to systematically investigate the global and local mechanisms by which vegetation types influence near-surface O_3_ concentrations from a street-view perspective. The main findings are as follows: (1) Early-summer O_3_ concentrations in Hangzhou exhibited significant spatiotemporal heterogeneity, showing an overall decreasing trend temporally and a dual spatial pattern of “higher in the center, lower in the periphery” and “higher in the northwest, moderately high in the southeast”. (2) At the global scale, the contribution of vegetation-type factors (0.001–0.011) is substantially lower than that of meteorological factors (0.041–0.437), with SAP and GAP showing relatively higher average contributions (0.006) and exerting positive and negative effects on O_3_ concentration, respectively. (3) At the local scale, the mean local importance of vegetation‑type factors is lower than that of meteorological factors and urban structure factors.But areas with high importance of vegetation-type factors largely overlapped with regions of high O_3_ concentrations, indicating that vegetation types play a significant regulatory role in O_3_ levels within core urban areas and industrial agglomerations. (4) High-importance areas of BD and ISP spatially overlapped to some extent with those of vegetation-type factors, whereas high-importance areas of meteorological factors and vegetation-type factors were clearly spatially separated. This suggests that vegetation types exhibit strong spatial coupling with BD and ISP in jointly regulating O_3_ concentrations, while meteorological factors dominate relatively independently in the urban periphery. The combination of segmented street-view imagery and DeepLabV3 + semantic segmentation enables efficient acquisition of micro-scale vegetation-type data. The Bayesian-optimized XGBoost model and the GWRF model can accurately capture the nonlinear relationships and spatial heterogeneity between vegetation types and O_3_ concentrations, providing a reliable technical approach for analyzing the regulatory effects of urban green spaces on O_3_. The study offers scientific evidence and practical references for precise O_3_ pollution control and optimized urban green space configuration.

## Supporting information

S1 File Supporting tables containing O_3_ concentration data (2014, 2017, 2021), input datasets for the Bayesian optimized XGBoost model (2014, 2017, 2021), input datasets for the GWRF model (2014, 2017, 2021), and feature local importance results (2014, 2017, 2021). (This file includes S1–S12 Tables as separate sheets/sections).
(ZIP)
